# Quantifying lung aeration in neonatal lambs at birth using lung ultrasound

**DOI:** 10.3389/fped.2022.990923

**Published:** 2022-09-28

**Authors:** Emily J. Pryor, Douglas A. Blank, Stuart B. Hooper, Kelly J. Crossley, Shiraz Badurdeen, James A. Pollock, Andrew V. Stainsby, Linda C. P. Croton, Dylan W. O’Connell, Christopher J. Hall, Anton Maksimenko, Daniel Hausermann, Peter G. Davis, Marcus J. Kitchen

**Affiliations:** ^1^The Ritchie Centre, Hudson Institute of Medical Research, Clayton, VIC, Australia; ^2^Department of Obstetrics and Gynecology, Monash University, Clayton, VIC, Australia; ^3^Monash Newborn, Monash Children’s Hospital, Clayton, VIC, Australia; ^4^Newborn Research Centre, The Royal Women’s Hospital, Parkville, VIC, Australia; ^5^School of Physics and Astronomy, Monash University, Clayton, VIC, Australia; ^6^ANSTO, Australian Synchrotron, Clayton, VIC, Australia

**Keywords:** lung ultrasound (LUS), neonate, respiratory distress at birth, lung aeration, computed tomography

## Abstract

**Background:**

Lung ultrasound (LUS) is a safe and non-invasive tool that can potentially assess regional lung aeration in newborn infants and reduce the need for X-ray imaging. LUS produces images with characteristic artifacts caused by the presence of air in the lung, but it is unknown if LUS can accurately detect changes in lung air volumes after birth. This study compared LUS images with lung volume measurements from high-resolution computed tomography (CT) scans to determine if LUS can accurately provide relative measures of lung aeration.

**Methods:**

Deceased near-term newborn lambs (139 days gestation, term ∼148 days) were intubated and the chest imaged using LUS (bilaterally) and phase contrast x-ray CT scans at increasing static airway pressures (0–50 cmH_2_O). CT scans were analyzed to calculate regional air volumes and correlated with measures from LUS images. These measures included (i) LUS grade; (ii) brightness (mean and coefficient of variation); and (iii) area under the Fourier power spectra within defined frequency ranges.

**Results:**

All LUS image analysis techniques correlated strongly with air volumes measured by CT (*p* < 0.01). When imaging statistics were combined in a multivariate linear regression model, LUS predicted the proportion of air in the underlying lung with moderate accuracy (95% prediction interval ± 22.15%, *r*^2^ = 0.71).

**Conclusion:**

LUS can provide relative measures of lung aeration after birth in neonatal lambs. Future studies are needed to determine if LUS can also provide a simple means to assess air volumes and individualize aeration strategies for critically ill newborns in real time.

## Introduction

During fetal development the future airways are liquid-filled, which is essential for normal lung development (1). At birth, this liquid must be cleared quickly in order to establish pulmonary gas exchange and facilitate a healthy transition from fetal to newborn life. Over 5% of all newborns require assistance to aerate their lungs, but the optimal type of assistance is unknown and likely varies between individuals (2). To optimize respiratory support in individual infants immediately after birth, real-time feedback is required to monitor the extent and progression of lung aeration. Imaging techniques that have been used to quantify lung aeration and liquid clearance include X-ray imaging (3) and computed tomography (CT) (4). While these imaging modalities provide detailed and accurate information on the degree of lung aeration, they expose the subject to ionizing radiation, making them inappropriate for repeated measurements in human infants. In contrast, lung ultrasound (LUS) does not utilize ionizing radiation, is quick, easy to perform at the bedside and the images are easy to interpret by clinicians (5–7). In addition, several studies have shown that LUS predicts the need for surfactant in preterm infants with respiratory distress syndrome (5, 8–12). However, it is unclear whether LUS can quantify relative lung aeration within specific lung regions immediately after birth and detect subtle changes over time.

**FIGURE 1:**
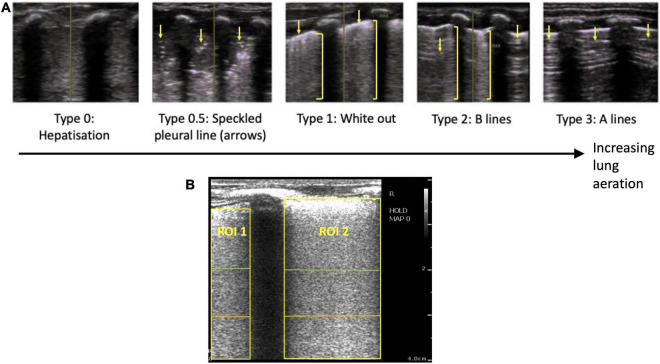
**(A)** Grading system used to describe the evolution of lung ultrasound images during the transition to air-breathing adapted from Raimondi et al. (10), as previously used by Blank et al. (7). Type 0: “hepatization,” the ultrasound beam does not encounter air, as it passes through liquid and soft tissue. The pleural line is either extremely thin or hypoechoic. Type 0.5: “speckled” pleural line, patchy hyperechoic appearance with poor definition and is not horizontal in orientation. This image is transiently visible in neonates after the initiation of breathing, but before the establishment of the pleural line. Type 1: “white-out lung,” associated with respiratory distress syndrome. Type 2: vertical “B lines” arising from the pleural line (brackets), with areas of horizontal A-lines (arrows). Type 3: horizontal “A-lines,” encountered when the US beam bounces between an aerated lung and the US transducer. **(B)** Example lung ultrasound image with two regions of interest (ROI; area below the pleural line, not including acoustic shadows) selected. A region of interest with a 2 cm depth is highlighted in green, a 3 cm depth is highlighted in orange, and a 4 cm depth is highlighted in yellow.

Unlike traditional ultrasound, LUS images are largely comprised of imaging artifacts, which change depending on the properties of the underlying lung tissue. In a well aerated lung, the presence of air below the pleura causes the ultrasound beam to bounce back and forth between the pleura and the transducer, which creates a series of pleural line echoes, termed A lines (13) (Figure 1A). If the lung is partially liquid-filled or deflated, the ultrasound beam can travel deeper into the lung *via* liquid or tissue “acoustic channels.” Here, it is thought to reverberate in the liquid, bouncing between aerated alveoli and creating a bright line of vertical echoes termed B lines (14). The number, type and intensity of B lines change depending on the relative amount of liquid vs. air in the lung (4) (Figure 1A).

Using a LUS grading system adapted from Raimondi et al. (10, 15), we have recently described the changes in LUS images in the first 24 h after birth in human infants, which was assumed to reflect progressive lung aeration and lung liquid clearance (6, 7). Before the onset of air-breathing, the liquid-filled lung efficiently conducts ultrasound, producing a true image of lung tissue that is categorized as Type 0 (Figure 1A). During the initiation of air breathing, the pleural line is patchy in appearance and the ultrasound image consists of speckled hyperechoic areas mixed with hypoechoic lung tissue, categorized as Type 0.5 (Figure 1A). Soon after birth, tightly packed B lines become visible, creating a “white out” image classified as Type 1 (Figure 1A). With increasing lung aeration, these B lines separate into distinct vertical lines and A lines begin to appear, which is categorized as Type 2 (6). When the lungs become well-aerated, which can take from minutes to hours after birth, B lines disappear and only A lines are visible, which is categorized as Type 3 (6, 7). These studies have demonstrated that changes in LUS images are associated with improving lung function and can predict the future need for surfactant therapy (5). However, it is not clear whether changes in lung aeration produce artifacts in LUS images that are consistent during lung inflation and deflation and whether the changes in LUS images are sensitive enough to detect small differences in lung aeration.

Analytical techniques that measure the brightness [mean pixel intensity (MPI)] of artifacts in LUS images have been utilized in adult preclinical (4) and clinical (16) models to estimate relative amounts of air and liquid in the lungs. However, pulmonary edema in pediatric and adult models (17) is fundamentally different to the liquid-filled airways at birth (1). Thus, it is unclear whether these analytical techniques are able to accurately monitor lung aeration in newborns at birth. One aim of this study was to determine if a qualitative LUS grading system (6, 7, 10) can accurately monitor lung aeration in newborn lambs. This was achieved by comparing LUS images to the gold standard for quantifying air, tissue and liquid volumes, high-resolution phase-contrast computed tomography (CT) scans (18). Using this imaging technique allowed for accurate calculations of lung volumes in the lung region being imaged by LUS. We also aimed to develop an analytical model that uses this grading system in addition to other aspects of LUS images, to quantify the volume of air in the lungs of newborn lambs.

## Materials and methods

### Animal ethics

All experimental procedures were approved by both Monash University’s Animal Ethics Committee and conducted in accordance with the National Health and Medical Research Council (NHMRC) Australian code of practice for the care and use of animals for scientific purposes (19).

### Experimental procedure

Near term fetal lambs (∼139 days of gestational age, term ∼148 days) were delivered and euthanized immediately with an overdose of sodium pentobarbitone before the onset of breathing. Lung development at this gestational age is equivalent to a near term to early term human infant. They were then intubated with a 4.5 mm cuffed endotracheal tube. A syringe was used to withdraw as much lung liquid from the lungs as possible, *via* the trachea, before 15 mL/kg of lung liquid (and normal saline if required to make up the difference) was then reintroduced *via* the endotracheal tube. This was done to standardize the amount of airway liquid volumes between animals, which can vary considerably (1). Care was taken to avoid any air from entering the lung during this process.

The lamb’s chest was shaved and it was secured upright in a custom-built frame for CT imaging of lambs. The frame was placed on the CT stage in experimental hutch 3 of the Imaging and Medical Beamline at the Australian Synchrotron. Fiducial markers were placed on both sides of the chest immediately cranial to the region imaged by ultrasound and used to identify regions imaged by LUS in the CT images. Imaging commenced approximately 1–4 h after the lamb was culled. LUS images were acquired as 3-s DICOM clips with a Phillips CX-50 ultrasound machine and an L3–12 linear transducer, with a frame rate of 15 images per second, a gain of 74 and harmonics turned off. LUS images and a CT scan were acquired prior to aeration, when the lungs were liquid-filled, before the endotracheal tube was connected to a small animal ventilator (4DMedical, Australia) (20). Nitrogen gas (rather than air or oxygen) was used to aerate the lungs to avoid oxygen diffusion out of the airways during imaging. The gradual loss of oxygen from the airways would reduce lung gas volumes during imaging, creating motion artifacts in the CT images. Aeration (with 100% nitrogen) was commenced using a static mean airway pressure of 15 cmH_2_O for approximately 2 min. The endotracheal tube was clamped, LUS images were acquired and a CT scan performed before the endotracheal tube was unclamped. The airway pressure was then increased by 5 cmH_2_O and the process was repeated until a maximum airway pressure of 50 cmH_2_O was reached. After this, the airway pressure was gradually decreased back to atmospheric pressure in 5–10 cmH_2_O increments, with airway reflooding expected to occur at low pressures. A CT scan was performed if a change was seen in the LUS image. Finally, as much gas as possible was suctioned out of the endotracheal tube using a 50 mL syringe, the endotracheal tube was clamped, and another LUS and CT scan was performed.

Each CT scan was comprised of 1,800 propagation-based phase contrast X-ray images acquired through 180 degrees of rotation using monochromatic synchrotron radiation tuned to 45 keV. The length of each CT scan was approximately 1 min. Propagation-based phase contrast was used to provide exceptional contrast between air and tissue/liquid in the lungs (18). The X-ray source-to-sample distance was ∼140 m and the sample-to-detector distance was 3.0 m. An Eiger2 X CdTe 2M-W (Dectris, Ltd., Switzerland) photon-counting detector was used, which provided a 311 mm wide by 38 mm field of view, and a pixel size of 75 μm, which was sufficient to fully resolve all but the smallest airways (alveoli) of the lungs. The limited height allowed only a section of the lamb’s chest to be imaged. The magnetic field strength of the beamline’s wiggler insertion device was set to 1.4 T and a 12 mm thick aluminum filter was placed before the sample to reduce the flux to the highly sensitive detector. At the conclusion of each imaging sequence, flat-field images were acquired to correct for variations in beam intensity.

### Computed tomography reconstruction and analysis

Before CT reconstruction, each projection image was pre-processed using the flat field images and a phase retrieval algorithm was applied (21) to remove the phase contrast effects and significantly boost the signal-to-noise ratio (18). A custom interpolation function was also used to remove vertical bands created by gaps between modules in the detector that otherwise produce strong CT ring artifacts. The CTs were then reconstructed using filtered back-projection with the XTRACT software (CSIRO, Australia).

Reconstructed CT scans were cropped to only include the region directly in line with the ultrasound probe, using the fiducial marker as a guide. To calculate air volumes, the gray values attributed to air (<0.15 m^–1^) were isolated in each reconstructed slice to produce binary images. CT scans were imported into 3D Slicer (Harvard University and National Institutes of Health, USA) and segmentation tools (draw, paint, fill between slices) were used to define the boundary of the lung. Within the lung segmentation, the threshold tool (with the given threshold used in the binary images) was used to differentiate air from tissue and liquid. The markup, fill between slices and logical operators (subtract) tools were then used to define regions of lung tissue and air within 1 cm of the pleural line. This depth was chosen as it excluded large vessels and airways that would interfere with volume calculations, and the ultrasound beam would be unlikely to penetrate deeper than 1 cm in a partially aerated lung (14, 22). The segment statistics tool was used to determine the number of air voxels within this segment, which was then used to calculate the proportion of air in the most superficial 1 cm region of the lungs (the number of air voxels divided by the total number of voxels in that lung region).

### Lung ultrasound image analysis

Lung ultrasound images were analyzed using the following techniques.

1 Qualitative scoring: LUS images were graded using a previously described grading scale (7, 10) (Figure 1A) by two blinded researchers (DB, EP). Where there was disagreement between the grade allocated by the two researchers, a third researcher (SB) was used as a tiebreaker.
2 Quantitative scoring: Rectangular regions of interest (ROIs; lung areas only) were defined manually below the pleural line and between acoustic shadows formed by the ribs, to a depth of 2, 3, and 4 cm (Figure 1B). Python packages SciPy and NumPy were used to calculate imaging statistics in each of these regions of interest for each frame in the 3-s ultrasound recordings, including:

• Mean pixel intensity (MPI)
• Coefficient of variation (CoV) of pixel intensity
• Fourier transform power spectral analyses along two orthogonal axes—(i) parallel to the pleural line and (ii) perpendicular to the pleural line. The axis parallel to the pleural line aimed to detect the strength of the B lines, while the axis perpendicular to the pleural line aimed to detect the strength of the A lines.

For each imaging statistic, a mean value for each ROI was calculated for each frame over the whole 3-s ultrasound clip. In ultrasound recordings with multiple ROIs, a weighted overall mean was calculated, where each ROI’s contribution was weighted based on the area of that ROI. To evaluate the relationship between the proportion of air in the lung and the imaging statistics identified above, the data were analyzed using:

1. all LUS images,
2. LUS images acquired during lung inflation and,
3. LUS images acquired during lung deflation.

Only type 1 and type 2 images were included in this analysis.

### Statistical analysis

We hypothesized that we would see a progressive increase in LUS type in each lung from type 0, 0.5, 1, 2, to 3 as we increased the inflation pressures incrementally, and then a decrease in LUS type from 3 back to 0 as inflation pressures were decreased. Therefore, *N* = 7 lambs would provide more than 100 paired observations between LUS and phase contrast CT measurements and be sufficient for statistical analysis.

Data were tested for normality using a Shapiro Wilk test. Descriptive statistics are presented as mean ± SEM if data were normally distributed, or median (interquartile range; IQR) if data were not normally distributed. A two-tailed *p*-value of *p* < 0.05 was considered statistically significant.

#### Qualitative scoring

The relationship between the proportion of air in the lungs, the LUS grade, and the mean airway pressure used were compared using a Kruskal-Wallis test (GraphPad Prism). The amount of volume loss required to cause a decrease in LUS grade was analyzed using a paired *t*-test (GraphPad Prism).

#### Quantitative scoring

The relationship between each imaging statistic and the proportion of air in the lungs, in type 1 and 2 images only, were compared using simple linear regression performed using the *statsmodel* package in Python. As the relationship between the area under the Fourier power spectra curve (parallel to the pleural line) and volume was found to be logarithmic, data were linearized on a log scale before performing linear regression. Separate models were developed to include all data points, inflation data points only, and deflation data points only.

#### Combining qualitative and quantitative scoring in a multivariate linear regression model

A multivariate linear regression model incorporating all imaging statistics from type 1 and 2 LUS images was produced using the *statsmodels* package in Python, with the proportion of air as the dependent variable. Stepwise backward elimination was used to select the most significant variables.

## Results

Paired CT and LUS images were obtained from a total of seven lambs (*N* = 7). From the CT analysis, the maximum proportion of air achieved in each lung was 63.8 ± 4.9% of total lung volume (combined air and liquid/tissue space).

### Qualitative scoring

**FIGURE 2:**
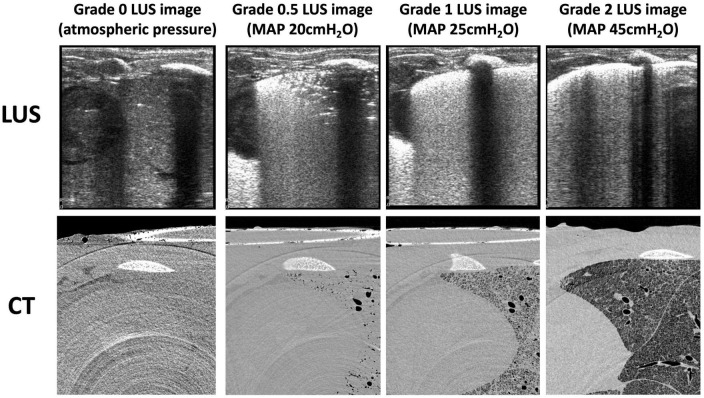
Example lung ultrasound images and an axial slice from the corresponding CTs of one animal during the inflation sequence. CT slices are grayscale (black represents air, light gray represents liquid or tissue, white represents bone).

**FIGURE 3:**
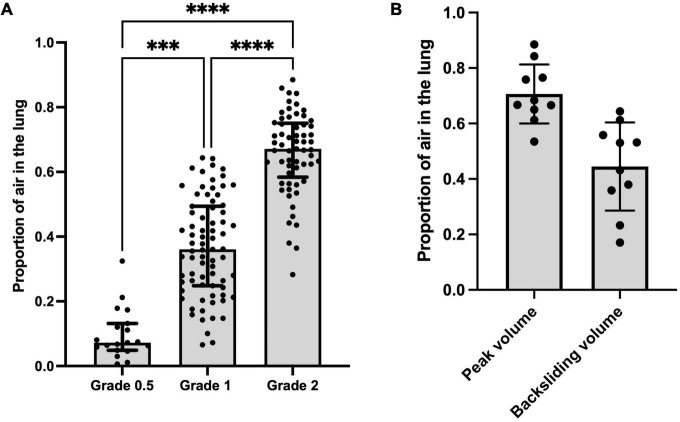
**(A)** Proportion of air vs. lung ultrasound grade at all data points. Kruskal-Wallis test; ^***^p < 0.001, ^*⁣*⁣**^p < 0.0001. **(B)** Proportion of air measured *via* CT at peak aeration pressure and proportion of air measured *via* CT when a decrease in LUS grade (from type 2 to type 1, termed “backsliding”) was first observed during the deflation sequence (mean of differences 0.26 ± 0.01; *p* = 0.12).

All lungs had a type 0 LUS image before ventilation onset (Table 1 and Figure 2). To achieve each successive LUS type required significantly higher airway pressures and was associated with a significantly higher proportion of air, although the range of measured volumes were very large for images classified as type 1 or 2 (Kruskal-Wallis test, *p* < 0.0001; Table 1 and Figures 2, 3A). During lung deflation, a decreasing airway pressure resulted in a visible decrease in LUS type (From type 2 to type 1) in 10 of 12 lungs, which was associated with a 26.2 ± 7.1% decrease from the maximum proportion of air (Figure 3B; paired *t*-test, *p* = 0.12). With suctioning, we were able to achieve a type 0 LUS image in one lung, which was associated with a proportion of air of 7.4%.

**TABLE 1:** Mean airway pressures (MAP) and proportions of air required to achieve each LUS grade.

LUS grade	Number of lungs (*n* = 14) in which this LUS grade was observed	MAP (cmH_2_O) required to first obtain this LUS grade (median, IQR)	Proportion of air (%) required to first achieve LUS grade (median, IQR)	Proportion of air (%) in all images at this LUS grade (median, IQR)
Type 0	14	0.0 (0.0–0.0)	0.0 (0.0–0.0)	0.0 (0.0–0.0)
Type 0.5	12	15.0 (14.0–15.0)	6.1 (3.4–7.9)	7.2 (4.9–13.2)
Type 1	14	20.0 (18.8–23.0)	20.7 (12.9–25.5)	36.1 (24.8–49.4)
Type 2	12	35.0 (30.0–38.8)	53.7 (45.0–65.5)	67.1 (58.4–75.0)
Type 3	0	N/A	N/A	N/A

### Quantitative scoring

**FIGURE 4:**
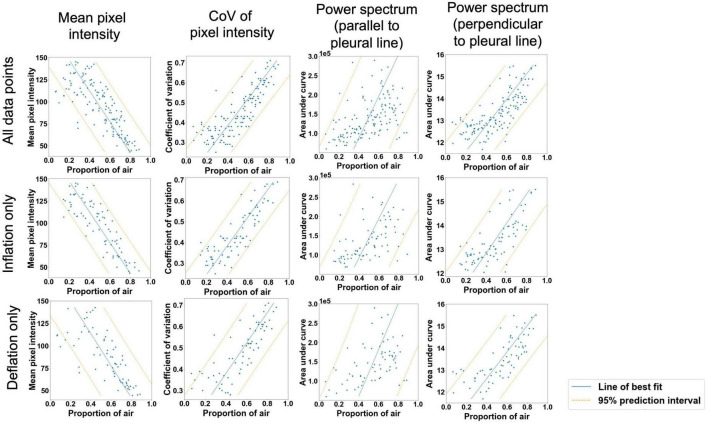
Moderately strong linear relationships (*p* < 0.001 for all variables) were observed between the proportion of air in the lungs and mean pixel intensity in the ultrasound lung spaces to a depth of 4 cm; the coefficient of variation of pixel intensity in the ultrasound lung spaces to a depth of 2 cm; and the area under the power spectral curve in the direction expected to identify B lines (parallel to the pleural line in Fourier space) and A lines (perpendicular to the pleural line in Fourier space). Blue line = simple linear regression model, orange lines = boundaries of 95% prediction interval.

There were highly significant linear associations between each of the four imaging statistics analyzed and the proportion of air in the lungs (Table 2 and Figure 4). In general, these relationships were stronger during lung inflation in comparison to during deflation.

**TABLE 2:** Simple linear regression models comparing quantitative LUS scoring methods and the proportion of air in the lungs (Figure 4).

	All data points	Inflation data points only	Deflation data points only
Mean pixel intensity (depth of 4 cm in LUS images)	*r*^2^ = 0.634, *p* < 0.001, 95% prediction interval ± 24.5%	*r*^2^ = 0.731, *p* < 0.001, 95% prediction interval ± 20.7%	*r*^2^ = 0.550, *p* < 0.001, 95% prediction interval ± 28.5%
CoV of pixel intensity (depth of 2 cm in LUS images)	*r*^2^ = 0.684, *p* < 0.001, 95% prediction interval ± 22.8%	*r*^2^ = 0.723, *p* < 0.001, 95% prediction interval ± 21.0%	*r*^2^ = 0.641, *p* < 0.001, 95% prediction interval ± 25.4%
Power spectral analysis, parallel to pleural line (depth of 4 cm in LUS images)	*r*^2^ = 0.219, *p* < 0.001, 95% prediction interval ± 35.8%	*r*^2^ = 0.234, *p* < 0.001, 95% prediction interval ± 35.0%	*r*^2^ = 0.184, *p* < 0.001, 95% prediction interval ± 38.3%
Power spectral analysis, perpendicular to pleural line (depth of 4 cm in LUS images)	*r*^2^ = 0.555, *p* < 0.001, 95% prediction interval ± 27.0%	*r*^2^ = 0.555, *p* < 0.001, 95% prediction interval ± 26.7%	*r*^2^ = 0.571, *p* < 0.001, 95% prediction interval ± 27.8%

### Combining qualitative and quantitative scoring in a multivariate linear regression model

The variables included in the final multivariate linear regression model were:

• CoV (*p* < 0.0001)
• LUS grade (*p* = 0.002)

**FIGURE 5:**
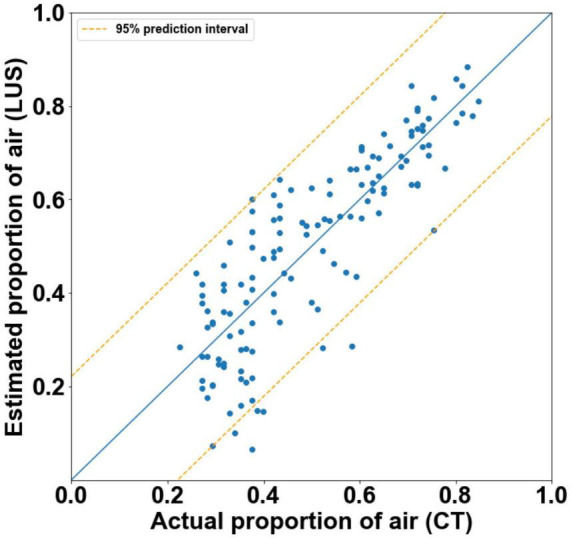
Actual proportion of air as measured by CT, vs. estimated proportions of air with 95% prediction intervals (orange) as calculated by final multivariate linear regression models (*r*^2^ = 0.71). The blue line represents the estimated proportions of air if the model was perfect (*r*^2^ = 1).

The final model was able to quantify the proportion of air in the lungs with moderate accuracy (Figure 5). It had an *r*^2^-value of 0.705 and a 95% prediction interval of ± 22.2%.

## Discussion

To optimize respiratory support after birth in individual infants, real-time feedback is required to monitor the extent and progression of lung aeration. While LUS can accurately estimate lung liquid volumes in adults with pulmonary edema (16, 17), neonates have much higher lung liquid volumes (35–40 mL/kg) (1) and regional variability in airway liquid at birth. This study has shown that LUS can provide an accurate, relative measure of lung gas volumes during lung aeration, using a variety of different approaches to analyze the LUS images.

We used a “gold standard” technique (high resolution phase contrast CT) for measuring regional air and lung tissue volumes to assess the capability of LUS to relative lung gas volumes. Using CT, we found that when the lungs were inflated to their maximum pressure (50 cmH_2_O), the proportion of total lung volume comprised of gas was 63.8 ± 4.9%, with the remainder comprised of tissue and liquid. Based on previous studies (23), this is notably less than would be expected in a fully aerated newborn lung at this gestational age (75–80%) (1). However, in our study, the higher relative tissue volume was expected due to tissue expansion resulting from the movement of airway liquid into lung tissue, which then increases tissue pressure (24). In contrast, previous estimates of airspace and tissue space volumes were obtained from histological sections from fetal lungs that had not undergone lung aeration (23) and so no airway liquid will have moved into lung tissue (25). Instead, much of the liquid will have been lost during tissue collection and during histological processing of the tissue, which involves both tissue dehydration and rehydration steps.

**FIGURE 6:**
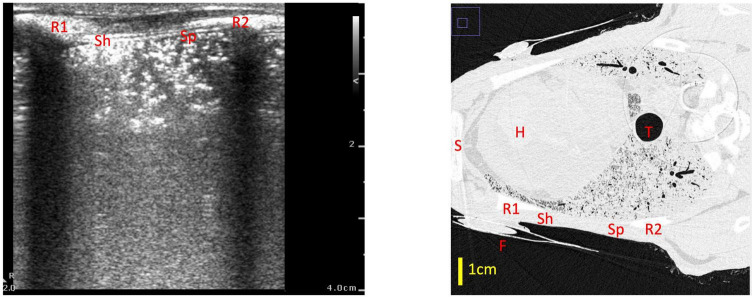
Example of a type 0.5 lung ultrasound image and a slice from the corresponding CT scan, with the medial rib (R1), lateral rib (R2) and fiducial (F) highlighted in both images. For reference, the heart (H), trachea (T) and sternum (S) are also labeled in the CT. The part of the ultrasound image closest to the medial rib (Sh) has a sharper pleural line which appears more superficially on the ultrasound image (at a depth of ∼0.5 cm), which corresponds with a better aerated portion of lung on CT—in this section, the ultrasound beam did not travel far before encountering a large, reflective block of air. The part of the ultrasound image which is closer to the lateral rib (Sp) has a more speckled pleural line, with small sections of the pleural line appearing at various depths from 0.5 to 2 cm. This corresponds with a more poorly aerated segment of lung on CT. In this section, the unaerated portions of lung form liquid/tissue channels which the ultrasound beam can travel through, until it gets reflected by small pockets of air which are present at various depths below the pleural line, creating the speckled appearance in the ultrasound image.

We found that using a qualitative LUS grading system (6, 10), a type 0 image was only observed when no (or very minimal) amounts of air were present in the lung. This indicates, with a high degree of confidence, that the lung region being imaged is almost completely liquid-filled if a type 0 image is observed. This is consistent with previous studies, where type 0 images were only observed in infants before the first breath was taken (7). Similarly, type 0.5 images were only observed in poorly aerated lungs and we found that they were associated with only 7.2% (median; IQR 4.9–13.2%) of the lung region being gas filled. With this image type (0.5), it is possible that liquid in the airways can form liquid channels through which the ultrasound beam can travel until it is eventually reflected by small pockets of aerated alveoli. As these aerated pockets are first present at varying depths below the pleural line, the ultrasound images contain a pleural line that appears speckled, with small portions of it appearing at various depths in the images (Figure 6). In any event, a type 0.5 image indicates that the underlying lung tissue is poorly aerated when acquired during initial lung aeration at birth, with the airways being mostly liquid-filled (7). As a type 0.5 image consistently indicates poor aeration with a narrow range of air/tissue proportions, we did not incorporate type 0.5 images into the models developed from imaging statistics.

While type 2 images were consistently associated with better aeration than type 1 images, the range of air/tissue proportions measured for both type 1 and 2 images was quite wide, with significant overlap between the two types (Figure 3A). This is an important finding, because type 1 images are being used in clinical research to predict infants requiring intubation after birth (5, 10) and to guide surfactant therapy. Indeed, in a recent clinical trial, infants < 32 weeks with a LUS score of ≥ 9 (approximately equivalent to a type 1 image in most of the 6 different lung regions imaged) soon after birth received early surfactant therapy, while those with a LUS score ≤ 8 did not receive surfactant (11). However, based on our CT data, it is quite possible that some infants with a LUS ≥ 9 may not have required surfactant therapy, whereas some with a score ≤ 8 could have benefited from surfactant therapy. We believe that a better model is required to distinguish between infants with well-aerated and poorly aerated lungs with a type 1 or 2 LUS appearance, in order to better predict infants at risk of deteriorating.

Analyzing the LUS images using alternative approaches were able to more accurately quantify the proportion of air in the lungs than the grading system in lungs with a type 1 or 2 appearance. The two analytical approaches that provided the closest association with the air/tissue proportion within the lung in a multivariate linear regression model were the CoV (*p* < 0.001) and LUS grade (*p* = 0.002). The final multivariate model had an *r*^2^-value of 0.71 and a 95% prediction interval of ± 22.2%, indicating that a combined analysis could be used to estimate the proportion of air from LUS images with moderate accuracy. However, as the simple linear regression model for the CoV has an *r*^2^-value of 0.68 and prediction intervals of ± 22.8%, incorporating the LUS grade into the model introduces a subjective measurement for only a small improvement in regression accuracy. As such, an analysis using CoV alone to estimate lung aeration from LUS images would be appear unbiased and most appropriate. We included all data points (i.e., both inflation and deflation data) in our model assessment, because it is unlikely that the inflation or deflation status of the lung will be known at the time of LUS imaging. As such, it would be difficult to determine whether an inflation or deflation model should be applied. For instance, the association between air/tissue proportion and area under the power spectra curve for inflation and deflation of the lung, depended upon the direction of analysis (i.e., parallel or perpendicular to the pleural line). The association was greatest in one direction during inflation and greater in the alternate direction during deflation.

No type 3 images were observed during this experiment, despite using very high inflation pressures of up to 50 cmH_2_O. While all the fetal lambs in this study were deceased, we have previously shown that this does not affect the rate, pattern or degree of lung aeration, other than requiring a mechanical ventilator to aerate the lung (3). Nevertheless, death will have prevented liquid from being cleared from lung tissue *via* the lymphatic system and so its continued presence in lung tissue likely prevented us from observing a type 3 image. In any event, a type 3 image on LUS is clearly indicative that the lungs are well aerated with little lung liquid remaining in the airways or in lung tissue. As type 3 images are often seen within minutes of birth in healthy newborn infants (7), it is likely that our lambs had more airway liquid at the onset of lung aeration than most newborn infants.

It is interesting that the predictive value of LUS images were considerably better during lung inflation compared with lung deflation. While the explanation for this finding is not known, it is possibly a function of hysteresis within the lung increasing the slope of the pressure-volume curve during lung deflation. This slope could also be accentuated and may differ across the lung due to the increased presence of liquid in lung tissue, which increases inter-alveolar tissue pressures and thereby must increase the collapsing pressure on alveoli. Indeed, inter-alveolar tissue is likely to contain considerably less liquid during lung inflation, than it will at the same airway volume during lung deflation. In retrospect, it would be very interesting to determine whether the predictive value of LUS during lung inflation and deflation could be improved with surfactant administration prior to the onset of lung aeration.

While our findings indicate that LUS images can predict the degree of lung aeration with moderate accuracy, our study has limitations. To eliminate motion artifacts in the CTs from the heart and breathing movements, we used deceased fetal lambs that, as stated above, likely prevented liquid clearance from lung tissue. Nevertheless, this meant that we were able to aerate the lungs slowly and progressively without concern for the animals’ gas exchange status and that progressive liquid clearance during the experiment did not alter the response to lung deflation that followed lung inflation. Additionally, we only used one type of ultrasound machine and transducer, and frequency settings were limited based on the machine. However, we would expect that our results would be similar using a different ultrasound machine, although we would need to confirm this. Finally, we were unable to distinguish between lung liquid retention and other conditions like atelectasis in the CT scans, although we expect little to no atelectasis during the inflation portion of the experiment. Nevertheless, it is possible that these conditions (such as respiratory distress syndrome or meconium aspiration) produce different patterns of artifacts on the LUS images, which could also affect the accuracy of the model.

## Conclusion

Our findings clearly demonstrate that LUS can provide a relative measure of lung aeration in deceased, near-term newborn lambs. Qualitative grading systems alone accurately reflected the proportion of gas in the lungs, particularly for type 0 and 0.5 LUS images. However, using more objective imaging statistics (such as the coefficient of variation of pixel intensity) to estimate the proportion of air from LUS appears most appropriate for type 1 and 2 images. Future studies are needed to determine if LUS can also provide a simple means to assess air volumes and individualize aeration strategies for critically ill newborns in real time.

## Data availability statement

The raw data supporting this conclusions of this article will be made available by the authors, without undue reservation.

## Ethics statement

Ethical review and approval was not required for the animal study because this experiment uses deceased animals scavenged from other experiments.

## Author contributions

MK, SH, DB, and EP conceived and designed the research and interpreted results of experiments, prepared figures and drafted the manuscript. MK, SH, DB, EP, KC, JP, AS, LC, DO’C, CH, AM, and DH performed experiments. EP and MK analyzed the data. All authors edited, revised, and approved the final version of the manuscript.

## References

[1] Hooper SB Harding R. Fetal lung liquid: a major determinant of the growth and functional development of the fetal lung. *Clin Exp Pharmacol Physiol.* (1995) 22:235–47. 10.1111/j.1440-1681.1995.tb01988.x 7671435

[2] Australian Institute of Health and Welfare [AIHW]. *Australia’s Mothers and Babies 2017—in Brief.* Canberra, ACT: AIHW (2019).

[3] Hooper SB Kitchen MJ Wallace MJ Yagi N Uesugi K Morgan MJ Imaging lung aeration and lung liquid clearance at birth. *Faseb J.* (2007) 21:3329–37. 10.1096/fj.07-8208com 17536040

[4] Corradi F Ball L Brusasco C Riccio AM Baroffio M Bovio G Assessment of extravascular lung water by quantitative ultrasound and CT in isolated bovine lung. *Respir Physiolo Neurobiol.* (2013) 187:244–9. 10.1016/j.resp.2013.04.002 23584050

[5] Badurdeen S Kamlin COF Rogerson SR Kane SC Polglase GR Hooper SB Lung ultrasound during newborn resuscitation predicts the need for surfactant therapy in very- and extremely preterm infants. *Resuscitation.* (2021) 162:227–35. 10.1016/j.resuscitation.2021.01.025 33548362

[6] Blank DA Kamlin COF Rogerson SR Fox LM Lorenz L Kane SC Lung ultrasound immediately after birth to describe normal neonatal transition: an observational study. *Arch Dis Child Fetal Neonatal Ed.* (2018) 103:F157–62. 10.1136/archdischild-2017-312818 28659360

[7] Blank DA Rogerson SR Kamlin COF Fox LM Lorenz L Kane SC Lung ultrasound during the initiation of breathing in healthy term and late preterm infants immediately after birth, a prospective, observational study. *Resuscitation.* (2017) 114:59–65. 10.1016/j.resuscitation.2017.02.017 28249708

[8] Razak A Faden M. Neonatal lung ultrasonography to evaluate need for surfactant or mechanical ventilation: a systematic review and meta-analysis. *Arch Dis Child Fetal Neonatal Ed.* (2020) 105:164. 10.1136/archdischild-2019-316832 31248960

[9] De Martino L Yousef N Ben-Ammar R Raimondi F Shankar-Aguilera S De Luca D. Lung ultrasound score predicts surfactant need in extremely preterm neonates. *Pediatrics.* (2018) 142:e20180463. 10.1542/peds.2018-0463 30108142

[10] Raimondi F Migliaro F Sodano A Ferrara T Lama S Vallone G Use of neonatal chest ultrasound to predict noninvasive ventilation failure. *Pediatrics.* (2014) 134:e1089–94. 10.1542/peds.2013-3924 25180278

[11] Rodriguez-Fanjul J Jordan I Balaguer M Batista-Muñoz A Ramon M Bobillo-Perez S. Early surfactant replacement guided by lung ultrasound in preterm newborns with RDS: the ULTRASURF randomised controlled trial. *Eur J Pediatr.* (2020) 179:1913–20. 10.1007/s00431-020-03744-y 32710304 PMC7378405

[12] Singh Y Tissot C Fraga MV Yousef N Cortes RG Lopez J International evidence-based guidelines on point of care ultrasound (POCUS) for critically ill neonates and children issued by the POCUS Working Group of the European society of paediatric and neonatal intensive care (ESPNIC). *Crit Care.* (2020) 24:65. 10.1186/s13054-020-2787-9 32093763 PMC7041196

[13] Lichtenstein DA Mauriat P. Lung ultrasound in the critically Ill neonate. *Curr Pediatr Rev.* (2012) 8:217–23. 10.2174/157339612802139389 23255876 PMC3522086

[14] Soldati G Copetti R Sher S. Sonographic interstitial syndrome: the sound of lung water. *J Ultrasound Med.* (2009) 28:163–74. 10.7863/jum.2009.28.2.163 19168766

[15] Raimondi F Migliaro F Sodano A Umbaldo A Romano A Vallone G Can neonatal lung ultrasound monitor fluid clearance and predict the need of respiratory support? *Crit Care.* (2012) 16:R220. 10.1186/cc11865 23151314 PMC3672599

[16] Corradi F Brusasco C Vezzani A Santori G Manca T Ball L Computer-aided quantitative ultrasonography for detection of pulmonary edema in mechanically ventilated cardiac surgery patients. *Chest.* (2016) 150:640–51. 10.1016/j.chest.2016.04.013 27130285

[17] Zong HF Guo G Liu J Bao LL Yang CZ. Using lung ultrasound to quantitatively evaluate pulmonary water content. *Pediatr Pulmonol.* (2020) 55:729–39. 10.1002/ppul.24635 31917899

[18] Kitchen MJ Buckley GA Gureyev TE Wallace MJ Andres-Thio N Uesugi K CT dose reduction factors in the thousands using X-ray phase contrast. *Sci Rep.* (2017) 7:15953. 10.1038/s41598-017-16264-x 29162913 PMC5698457

[19] National Health and Medical Research Council [NHMRC]. *Australian Code of Practice for the Care and use of Animals for Scientific Purposes.* Canberra, ACT: National Health and Medical Research Council (2013).

[20] Kitchen M Habib A Fouras A Dubsky S Lewis R Wallace M A new design for high stability pressure-controlled ventilation for small animal lung imaging. *J Instrum.* (2010) 5:T02002. 10.1088/1748-0221/5/02/T02002

[21] Paganin D Mayo SC Gureyev TE Miller PR Wilkins SW. Simultaneous phase and amplitude extraction from a single defocused image of a homogeneous object. *J Microsc.* (2002) 206:33–40. 10.1046/j.1365-2818.2002.01010.x 12000561

[22] Mohanty K Blackwell J Egan T Muller M. Characterization of the lung parenchyma using ultrasound multiple scattering. *Ultrasound Med Biol.* (2017) 43:993–1003. 10.1016/j.ultrasmedbio.2017.01.011 28318888

[23] Alcorn DG Adamson TM Maloney JE Robinson PM. A morphologic and morphometric analysis of fetal lung development in the sheep. *Anat Rec.* (1981) 201:655–67. 10.1002/ar.1092010410 7340570

[24] Miserocchi G Poskurica BH Del Fabbro M. Pulmonary interstitial pressure in anesthetized paralyzed newborn rabbits. *J Appl Physiol (Bethesda Md 1985).* (1994) 77:2260–8. 10.1152/jappl.1994.77.5.2260 7868443

[25] Hooper SB Te Pas AB Kitchen MJ. Respiratory transition in the newborn: a three-phase process. *Arch Dis Child Fetal Neonatal Ed.* (2016) 101:F266–71. 10.1136/archdischild-2013-305704 26542877

